# Treatment Efficacy of Plasmapheresis Versus Intravenous Immunoglobulin in Guillain-Barré Syndrome Management: A Systematic Review

**DOI:** 10.7759/cureus.57066

**Published:** 2024-03-27

**Authors:** Sanath Savithri Nandeesha, Alousious Kasagga, Chnoor Hawrami, Erica Ricci, Kirubel T Hailu, Korlos Salib, Samia Butt

**Affiliations:** 1 Internal Medicine, California Institute of Behavioral Neurosciences & Psychology, Fairfield, USA; 2 Pathology, California Institute of Behavioral Neurosciences & Psychology, Fairfield, USA; 3 Pediatric Surgery, California Institute of Behavioral Neurosciences & Psychology, Fairfield, USA; 4 Anesthesiology, California Institute of Behavioral Neurosciences & Psychology, Fairfield, USA; 5 Internal Medicine, Afet Speciality Clinic, Addis Ababa, ETH; 6 Internal Medicine, St. Mary El Zaytoun, Cairo, EGY; 7 Research, California Institute of Behavioral Neurosciences & Psychology, Fairfield, USA

**Keywords:** guillain-barré syndrome (gbs), guillain-barre syndrome (gbs), ivig treatment, therapeutic plasma exchange (tpe), intravenous immunoglobulins (ivig), therapeutic plasmapheresis

## Abstract

Guillain-Barré syndrome (GBS) is a rare and debilitating autoimmune disorder that affects the peripheral nervous system. Although the exact etiology of GBS is still unknown, it is thought to be triggered by a preceding gastrointestinal infection in most of the cases. Clinical manifestations include limb weakness, areflexia, and sensory loss that can further progress to neuromuscular paralysis affecting the respiratory, facial, and bulbar functions. Both plasmapheresis (PE) and intravenous immunoglobulin (IVIG) have shown effectiveness in the treatment of GBS, but it is still unclear which treatment approach is superior in terms of therapeutic efficacy. This systematic review acts per Preferred Reporting Items for Systematic Reviews and Meta-Analysis (PRISMA) 2020 guidelines. For appropriate studies and research, we searched PubMed, PubMed Central (PMC), Medical Literature Analysis and Retrieval System Online (MEDLINE), Science Direct, and Google Scholar. Screening of articles was performed based on relevance and inclusion and exclusion criteria. To check for bias, we used relevant quality appraisal tools. Initially, we found 2454 articles. After removing duplicates and irrelevant papers, we finalized 31 studies based on titles, abstracts, and reading entire articles. We excluded 14 studies because of poor quality; the remaining 17 papers were included in this review. IVIG is equally efficacious as PE in improving primary outcomes and secondary outcomes. IVIG showed a slight advantage over PE in reducing the need for mechanical ventilation (MV) and hospital stay duration. However, in children, PE demonstrated a slight edge in improving secondary outcomes. PE was associated with a slightly higher risk of adverse events and post-treatment worsening symptoms compared to IVIG. IVIG is considered more user-friendly with a significantly lower patient discontinuation rate than PE. IVIG treatment was found to be significantly more expensive than PE.

## Introduction and background

Guillain-Barré syndrome (GBS) is a rare and debilitating autoimmune disorder that affects the peripheral nervous system. Research shows that it has a global incidence rate of one to two per 100000 people yearly and affects people of all age groups [1]. GBS can occur in all age groups but is slightly more common in males than in females [2]. About 20% of patients experience GBS-related mortality or severe disability [3].

Although the exact etiology of GBS is still unknown, it is thought to be triggered by a preceding infection in most cases, most commonly respiratory or gastrointestinal infections. Seventy percent of GBS cases start one to three weeks following an acute infectious process. *Campylobacter jejuni, Mycoplasma pneumonia, Haemophilus influenzae, Cytomegalovirus, Epstein-Barr virus, and Influenza virus* are the organisms that are believed to be involved [3,4]. Several mechanisms have been proposed to explain the pathogenesis of GBS, with molecular mimicry playing a central role. *C. jejuni *possesses a lipooligosaccharide (LOS) in its outer membrane that shares structural similarities with gangliosides, components of the peripheral nerves [2]. This molecular mimicry can lead to cross-reactivity, where antibodies generated against the LOS during a *C. jejuni* infection mistakenly attack the gangliosides, causing nerve damage and the clinical manifestations of GBS. In addition to infectious triggers, non-infectious factors have also been implicated in the pathogenesis of GBS. These include vaccination, surgery, and trauma [5]. However, the exact mechanisms by which these factors contribute to GBS remain unclear.

Clinical manifestations include limb weakness, areflexia, and sensory loss that can further progress to neuromuscular paralysis affecting the respiratory, facial, and bulbar functions. Symptom severity peaks in two to four weeks [4]. There are several subtypes of GBS, each with its characteristics. The most common subtypes include acute inflammatory demyelinating polyneuropathy (AIDP): this is the most common subtype of GBS, accounting for about 70% of cases. AIDP is characterized by a rapid progression of symptoms, often reaching their peak within two to four weeks. Miller-Fisher syndrome: This is a less common subtype of GBS that affects the nerves in the face, eyes, and balance system. Miller-Fisher syndrome is characterized by weakness of the face and eyes, as well as difficulty with balance and coordination [5]. Acute motor axonal neuropathy (AMAN): This subtype of GBS affects the motor nerves, leading to weakness in the arms and legs. AMAN is more common in Asia than in other parts of the world. Acute motor and sensory axonal neuropathy (AMSAN): This subtype of GBS affects both the motor and sensory nerves, causing weakness, numbness, and tingling in the arms and legs. AMSAN is less common than AMAN. Cranial nerve GBS: This subtype of GBS affects the nerves in the head and neck. Cranial nerve GBS can cause a variety of symptoms, including weakness of the face and eyes, difficulty swallowing, and difficulty speaking. The most frequently affected nerve is the facial nerve, which is followed by the involvement of the extraocular muscles and lower cranial nerve. Autonomic GBS: This subtype of GBS affects the nerves that control the autonomic nervous system. Autonomic GBS can cause a variety of symptoms, including changes in heart rate, blood pressure, and bladder function [6].

Plasmapheresis (PE) and IVIG are both commonly used immunomodulation therapies in the management of GBS [7]. PE, also known as plasma exchange, is a procedure that removes harmful antibodies from the patient's blood. During PE, a portion of the patient's blood is extracted and passed through a machine that separates the plasma, which contains the antibodies, from the red blood cells and white blood cells. The red blood cells and white blood cells are then reintroduced into the patient's bloodstream, while the antibody-laden plasma is discarded. PE is typically administered for three to five sessions over one to two weeks. The number of sessions and the frequency of treatment may vary depending on the severity of the patient's GBS. Intravenous immunoglobulin (IVIG) involves administering a high dose of antibodies produced by healthy individuals to the patient. These antibodies help to suppress the patient's immune system and prevent it from attacking the nerves. IVIG is typically administered intravenously for five days [8]. Both PE and IVIG have shown effectiveness in the treatment of GBS, but it is still unclear which treatment approach is superior in terms of therapeutic efficacy [9].

This review aimed to compare the effectiveness of PE and IVIG in the treatment of GBS by analyzing relevant studies and synthesizing the findings. The systematic review findings will contribute valuable insights into the optimal treatment approach for GBS and inform clinical decision-making.

## Review

Methods

This systematic review follows the guidelines and principles of the Preferred. Reporting Items for Systematic Reviews and Meta-Analysis (PRISMA) 2020 [10].

Search Strategy

PubMed, PubMed Central (PMC), Medical Literature Analysis, Retrieval System Online (MEDLINE), Science Direct, and Google Scholar were used exclusively as research databases and search engines to conduct this systemic review. The research utilizes GBS, PE, and IVIG. We used the Boolean terms "AND" and "OR" to combine the relevant concepts with specific keywords, as shown in Table 1.

**Table 1:** MeSH strategy IVIG, intravenous immunoglobulin; GBS, Guillain-Barre syndrome; PE, plasmapheresis

Keywords	Search Builder
GBS	("Guillain-Barre Syndrome"[Mesh]) OR (“Guillain-Barre Syndrome/drug therapy"[Mesh] OR "Guillain-Barre Syndrome/therapy"[Mesh] )
PE	"Plasmapheresis"[Mesh]) OR "Plasma Exchange"[Mesh])
IVIG	"Immunoglobulins, Intravenous/therapeutic use"[Mesh]

Similarly, the same concepts were used as the keywords to create the following Medical Subject Headings (MeSH) strategy. We Selected subheadings like drug therapy and therapeutic use. The most recent search was conducted on October 29, 2023. The results are shown in Table 2.

**Table 2:** Advanced search strategy

Advanced search strategy	(((((("Guillain-Barre Syndrome"[Mesh]) OR (“Guillain-Barre Syndrome/drug therapy"[Mesh] OR "Guillain-Barre Syndrome/therapy"[Mesh] OR "Miller Fisher Syndrome"[Mesh] OR "Miller Fisher Syndrome/therapy"[Mesh])) AND "Plasmapheresis"[Mesh]) OR "Plasma Exchange"[Mesh]) AND "Immunoglobulins, Intravenous/therapeutic use"[Mesh]))

Screening of Articles and Data Extraction

Two reviewers (Sanath Savithri Nandeesha and Alousious Kasagga) independently screened titles and abstracts to determine their eligibility. All the relevant articles were collected, and duplicates were eliminated. Then, the relevant papers were screened based on title, abstract, and full-text reading. Finally, 17 research studies were selected and subjected to quality assessment. We completed the comprehensive search after manually reviewing the reference lists of included articles. We then extracted data from the selected studies. The following variables were analyzed using the standardized recording tool: study design, number of participants, baseline participant characteristics, type of fasting and duration of treatment, mean follow-up in each participant group, study outcomes, and funding source (pharmaceutical company or not).

Inclusion Criteria

Included randomized controlled trials (RCTs), observational studies (cohort studies and case-control studies), and cross-sectional studies comparing PE and IVIG as treatments for GBS were included. Studies involving patients diagnosed with GBS regardless of age or gender were considered. Studies that evaluated the use of PE or IVIG as a primary treatment for GBS and studies that reported clinical efficacy outcomes (e.g., improvement in muscle strength, duration of hospitalization, and respiratory support required) were included in the review.

Exclusion Criteria

Case reports, case series, reviews, and studies with insufficient data or did not report relevant clinical outcomes were excluded. Duplicate publications or multiple reports of the same study were excluded to avoid redundancy. Studies in languages other than English and published more than 35 years ago were excluded from this review.

Quality Appraisal

This systematic review included randomized trials, cohort studies, and cross-sectional studies. Quality appraisal tools such as the Newcastle-Ottawa Scale, Journal of Biomedical Informatics (JBI) checklist, and Critical Appraisal Skills Programme (CASP) checklist were applied to assess the risk of bias during the selection of papers as shown in Table 3 and Table 4 [11-13]. Only those articles that met more than 70% of the criteria were chosen.

**Table 3:** A quality appraisal of cohort and cross-sectional studies NOS, Newcastle-Ottawa Scale; JBI, Journal of Biomedical Informatics

Author	Type of Study	Quality Appraisal Tool	1	2	3	4	5	6	7	8
Chara et al., 2014 [14]	Cohort	Newcastle-Ottawa Scale	+	+	+	+	+	+	+	+
Rodprasert et al., 2014 [15]	Cohort	Newcastle-Ottawa Scale	+	+	+	+	+	+	+	?
Saad et al., 2016 [16]	Cohort	Newcastle-Ottawa Scale	+	+	+	+	+	+	+	+
El-Bayoumi et al., 2011 [17]	Cohort	Newcastle-Ottawa Scale	+	+	+	+	+	+	+	+
Mallick et al., 2019 [18]	Cohort	Newcastle-Ottawa Scale	+	+	+	+	+	+	+	+
Beydoun et al., 2020 [19]	Cohort	Newcastle-Ottawa Scale	+	+	+	+	+	+	+	+
Ashalatha, 2002 [20]	Cohort	Newcastle-Ottawa Scale	+	+	+	+	+	+	+	+
Kishore et al., 2014 [21]	Cohort	Newcastle-Ottawa Scale	+	+	+	+	+	+	+	+
Ravasio et al., 1995 [22]	Cohort	Newcastle-Ottawa Scale	+	+	+	+	+	+	+	+
Sudulagunta et al., 2015 [23]	Cohort	Newcastle-Ottawa Scale	+	+	+	+	+	+	+	+
Anwer et al., 2020 [24]	Cross-Sectional	JBI Checklist	+	+	+	+	+	?	+	+

**Table 4:** Quality appraisal of clinical trials CASP, Critical Appraisal Skills Programme; RCT, randomized clinical trial

Author	Type of Study	Quality Appraisal Tool	1	2	3	4	5	6	7	8	9	10	11
Sandoglobulin Guillain-Barre Syndrome Trial Group. 1997 [25]	RCT	CASP Checklist	+	+	-	+	+	+	+	+	+	+	?/-
Kuwabara et al., 2001 [26]	RCT	CASP Checklist	+	+	+	+	+	+	+	+	+	+	+
Ye et al., 2015 [27]	RCT	CASP Checklist	+	+	+	+	+	+	+	+	+	+	+
Elahi et al., 2019 [28]	Non-RCT	CASP Checklist	+	+	+	-	?/-	+	+	+	+	+	+
Asghar et al., 2015 [29]	RCT	CASP Checklist	+	+	+	+	+	+	+	+	+	+	+
Van der Meche et al., 1992 [30]	RCT	CASP Checklist	+	+	+	+	+	+	+	+	+	+	+

The Newcastle-Ottawa cohort checklist is a valuable tool for evaluating the quality of cohort studies. It consists of eight key criteria that must be considered when assessing the validity of a study. These criteria include the representativeness of the exposed cohort, the selection of the non-exposed cohort, the ascertainment of exposure, and the demonstration that the outcome of interest was not present at the start of the study. Additionally, the checklist evaluates the comparability of cohorts based on design or analysis, the assessment of outcome, the adequacy of follow-up, and whether follow-up was long enough for outcomes to occur. Adhering to these criteria ensures the reliability and credibility of cohort studies [11].

The JBI critical appraisal checklist for analytical cross-sectional studies provides a comprehensive framework for evaluating the quality of research in this study design. Key considerations include the clarity of inclusion criteria, detailed descriptions of study subjects and settings, valid and reliable measurement of exposures and outcomes, identification and handling of confounding factors, and appropriate statistical analysis [12].

The CASP checklist is a valuable tool for evaluating the quality of research studies. It prompts users to consider key aspects of a study, such as the clarity of the research question, randomization of participants, and blinding of investigators. These criteria help ensure that the study is rigorous and its results are reliable. By assessing factors like the similarity of study groups and the reporting of intervention effects, researchers can determine the validity of the study's findings. Ultimately, the CASP checklist aids in making informed decisions about the applicability and value of research findings to specific populations and contexts [13].

Results

**Figure 1:**
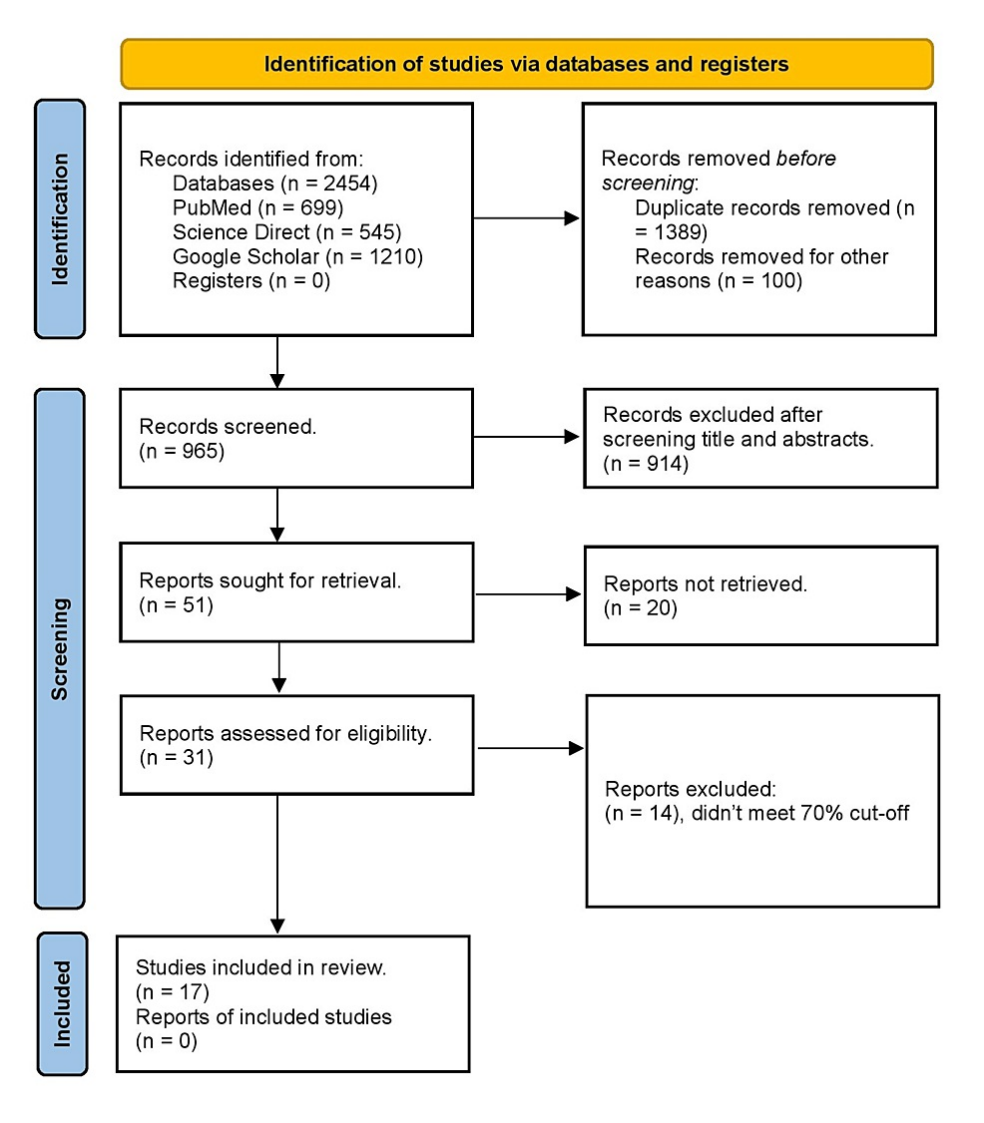
PRISMA flowchart PRISMA, Preferred Reporting Items for Systematic Reviews and Meta-Analysis

We searched databases electronically to look for relevant studies. Initially, we found 2454 articles related to our topic. Afterward, 1389 duplicates were removed. This number was further reduced to 31 after screening based on inclusion/exclusion criteria and relevant title, abstract, and full-text reading. Finally, the quality assessment tools assessed the bias in the studies. Ultimately, we finalized 17 articles and removed the remaining articles due to poor quality. The search strategy used to conduct this review is shown in a PRISMA flowchart in Figure 1.

The summary of the final 17 articles is displayed in Table 5.

**Table 5:** Summary of selected studies PE, plasmapheresis; IVIG, intravenous immunoglobulin; GBS, Guillain-Barré syndrome; MV, mechanical ventilation; RCT, randomized clinical trial; PLEX, plasma exchange; PICU, pediatric intensive care unit; TPE, therapeutic plasmapheresis

Author and Year of Publication	Type of Study	Intervention		Patient Characteristics		Result	Conclusion
Sandoglobulin Guillain-Barre Syndrome Trial Group, 1997 [25]	RCT	Five 50 mL/kg exchanges over 8-13 days	0.4 g/kg daily for 5 days	379	NA	In the 121 PE group patients, the mean improvement was 0·9 (SD 1·3), while in the 130 IVIg group patients, it was 0·8 (1·3). For this primary outcome, there were no significant differences between the groups. The secondary outcome measure also did not show a statistically significant difference between any of the treatment groups.	PE and IVIg had equivalent efficacy in improving both primary and secondary outcome
Chara et al., 2014 [14]	Cohort study	4 PE for 10-14 days	0.4 g/kg/day for 5 days	41	37.4±9.2 years mechanically ventilation adults	The average hospital stay lasted 45.3±9.2 days. The IVIG group's hospital stay was shorter than the PE group (p=0.03). In the IVIG group, the MV weaning occurred more quickly than in the PE group (p=0.01). Additionally, the IVIG group experienced a faster start to motility recovery than the PE group (p=0.04).	MV weaning and precocious recovery were significantly better in the IVIG group compared to the PE group
Rodprasert et al., 2014 [15]	Cohort study	Plasma exchange was to exchange 200 to 250 mL of plasma per kg of body weight in 3-5 sessions within 7 to 14 days	0.4 g/kg per day of immune globulin during the 5 subsequent days	40	NA	63% of PE patients improved functional grades after a month, compared to 57% in the IVIG group, with no significant difference in mean functional scale between treatments.	Concludes that, IVIG is a practical, safe, and effective treatment for GBS, But IVIG is more expensive.
Kuwabara et al., 2001 [26]	Clinical trial	50 mL/kg plasma 4-8 times daily	400 mg/kg/day for 5 days	24 IgG anti-GM1-positive subgroup of GBS patients	NA	Among, IgG anti-GM1-positive subgroup of GBS patients, patients treated with IVIG had a significantly lower Hughes score at 1, 3, and 6 months after disease onset (P=0.03) and a greater likelihood of regaining independent mobility at 6 months (P (logrank)=0.044). CMAP total scores at 6 months were higher in the IVIG group (P=0.07).	Treatment with IVIG therapy may be more effective than PE for GBS patients who are in the IgG anti-GM1-positive subgroup.
Saad et al., 2016 [16]	Cohort study	200-250 mL/kg was administered over 7-10 days	0.4 g/kg/day for 5 days	62	8.0±4.7 years	Mean duration of hospitalization in cases treated with PE (group 2) was significantly shorter than the time required for other treatment (group 1) (p<0.01). In group 1 treated with IVIG, the number of patients requiring MV was significantly higher (p<0.001). Patients treated with PE had a significantly higher complete recovery than the other group (p<0.001).	PE is the best treatment modality for GBS since it reduces the duration of hospital stay and speeds up the recovery of those children.
El-Bayoumi et al., 2011 [17]	Cohort study	One volume of PE per day	0.4 g/kg/day for 5 days	41 children with GBS requiring MV	49-143 months	Children in the PE group had a shorter duration of MV (median: 11 days, IQR: 11.0 to 13.0) compared to the IVIG group (median: 13 days, IQR: 11.3 to 14.5), p=0.037. Children in the PE group tended to have a shorter stay in the PICU (p=0.094). A total of 20/21 (95.2%) and 18/20 (90%) children in the PE and IVIG groups were able to walk without assistance within four weeks of PICU discharge (p=0.606).	In children with GBS requiring MV, PE is superior to IVIG regarding the duration of MV but not PICU stay or the short-term neurological outcome
Ye et al., 2015 [27]	Clinical trial	Five 50 mL/kg PEs	0.4 g/kg/day for 5 days	64	NA	The nerve function improved better in PE group than in the IVIG group. The treatment effectiveness in the PE and IVIG groups after 2 weeks were 96% and 79%, respectively. PE and IVIG significantly reduced blood immunoglobulins IgG, IgA, IgM, C3, and C4 in GBS patients, but they were significantly lower in the PE group than in the IVIG group.	PE therapy can efficiently reduce symptoms and aid in patients' early rehabilitation, which has a more profound therapeutic effect.
Elahi et al., 2019 [28]	Clinical trial	One-volume PE per day	0.4 g/kg/day for 5 days	78	6.64±3.06 years	Compared to children who received IVIG (4.97±2.84 days), children who received plasma exchange (9.45±4.59 days) had a significantly longer length of stay in the ICU (p-value <0.001, 95% CI -6.23 to -2.73). Similarly, children who received plasma exchange had a significantly longer mean ventilator stay (7.33±3.44 days) than children who received IVIG (2.01±0.01 days) (p-value <0.001, -7.91 to -2.74).	The outcome of IVIG was found to be better than that of plasma exchange in treating children with GBS.
Anwer et al, 2020. [24]	Cross-sectional study	NA	NA	34	9±3 years	The study found PE had the highest cure rate (100%), while IVIG had a modest cure rate (64%). However, 16% of IVIG patients left against medical advice, and 20% died.	PE is better than other treatment options.
Mallick et al, 2019. [18]	Cohort study	4 PE for 10 days every alternate day	0.4 g/kg/day	49	37.4±9.2 years	The IVIG and PE groups had an average ICU stay of 20±19.10 days and 46.60±30.02 days, respectively. Compared to the PE group, the IVIG group's ICU stay was significantly shorter (p=0.001). In comparison to patients receiving PE, those receiving IVIG were statistically significantly earlier to wean themselves off of MV (p=0.002). Additionally, the IVIG group outperformed the PE group in terms of M/V duration (P=0.002), tracheostomy need (p=0.005), and overall survival rate (p=0.007).	IVIG reduced the duration of hospital stay and hastened the recovery of patients with GBS.
Beydoun et al, 2020. [19]	Cohort study	NA	NA	6642	NA	Patients who underwent TPE stayed in the hospital for an average of 7.5 days longer than those receiving IVIG; additionally, their hospitalization expenses increased by an average of $46000 and their odds ratio for in-hospital death increased by 2.78.	TPE may be associated with poorer healthcare utilization outcomes vs. IVIG.
Ashalatha, 2002. [20]	Cohort study	100 mL plasma/kg body weight	0.4 g/kg/day	97	35.17 in PLEX, 33.76 in IVIG	27 patients (56.3%) in the PE group and 37 patients (75.5%) in the IVIG group saw improvements of one or more functional grades in 4 weeks (28 days); the difference was 19.2% (p<0.05). In the immune globulin group, a significantly greater percentage of patients achieved the primary outcome. The mean length of stay in the hospital was 26 days for the PE group and 16 days for the IVIG group (p=0.005). The IVIG group experienced a 28 day reduction in the median time to regain independent locomotion compared to the PE group (p=0.070).	IVIG gave better outcomes in 4 weeks and fewer complications when compared to PE.
Asghar et al, 2015. [29]	RCT	NA	NA	36 patients	37±15	Significant improvement of mean disability score was observed in both groups (p<0.0005). Comparison between the 2 groups in terms of mean improvement in disability scores showed significant improvement at 4 weeks (p<0.05) in the IVIG group when compared to the PE group. Further observation at 12 weeks and 6 months showed no significant difference (p>0.05) in mean improvement between the two groups. There was no significant difference in the need for MV between the 2 groups (p>0.05)	Significant short-term improvement was observed in the IVIG group at 4 weeks of treatment; however, no significant difference in therapeutic outcome was observed between the two groups on further follow-up of 6 months.
Kishore et al, 2014. [21]	Cohort study	200-250 mL of plasma per kg weight in five sessions (40-50 mL/kg per session) within 7-14 days	0.4 g/kg body weight daily	90 patients	Mean ages were 30.862 for IVIG and 35.737 years for PE	PE showed a slight superiority over IVIG in improving disability grading for both the entire group and respiratory patients, with a Z-value of 2.329 and a p-value of 0.000. The duration of illness at presentation was longer in the IVIG group (8.4483 days) compared to the PE group (6.442 days). The cost of PE is approximately $1300, whereas IVIG costs around $2400.	PE was found to be associated with more improvement and also cheaper in comparison to IVIG.
Emilia-Romagna Study Group on Clinical and Epidemiological Problems in Neurology, Italy Ravasio et al, 1995. [22]	Cohort study	PE (mean number of sessions: 5.3, range: 2-15; mean plasma exchange per session: 1900 mL)	0.4 g/kg/d	50	Age: 54.42±20.25 years	The study found that out of 20 patients treated with IVIG and 16 patients who underwent PE as first choice therapy, 9 improved, 4 recovered, and 4 remained unchanged at the end of the month.	No significant differences in terms of clinical outcome or electro-physiological features between treatments.
Van der Meche et al, 1992. [30]	RCT	200-250 mL/kg of PE for 5 sessions within 7–14 days	0.4 g/kg/day for 5 subsequent days	150	47.5±19.2 years	In the study, 34 percent of patients treated with plasma exchange showed an improvement in strength by one grade or more, compared to 53 percent of those treated with immune globulin, resulting in a 19 percent difference (P-value=0.024). The median time to improvement by one grade was 41 days with plasma exchange and 27 days with immune globulin therapy (P=0.05). Notably, the immune globulin group had fewer complications and a reduced need for artificial ventilation.	The primary outcome measure was achieved by a notably higher number of patients in the IVIG group. In the IVIG group, there was a lower frequency of MV requirements and complications.
Sudulagunta et al, 2015. [23]	Cohort study	200-250 mL/kg of plasma (total) over 5 to 8 cycles on a daily or alternate day basis	0.4 g/kg daily for 5 consecutive days.	1166 (962)	42.8±24 years (range from 0-85)	The mean MRC sum score for the IVIG group was 19.62±8.20 upon admission and 40.10±14.80 upon discharge (p<0.0001). Similarly, for the plasma exchange group, the mean MRC sum score was 19.42±11.16 upon admission and 40.64±15.80 upon discharge (p<0.0001). Additionally, patients in the plasma exchange group experienced a statistically significant increase in the length of their hospital stay compared to the IVIG group (p=0.001). However, there was no statistically significant difference in the occurrence of complications between the two groups.	The study found no significant difference in follow-up and outcome at discharge between the IVIG and plasma exchange groups, but a significantly longer hospital stay was observed in the plasma exchange group compared to the IVIG group. Additionally, IVIG treatment was found to be more expensive.

The systematic search identified 17 studies comparing PE versus IVIG in which efficacy was measured by improvement on established disability scales (Hughes disability scale mostly). Out of 17 studies, six were RCTs, 10 were cohort studies, and one cross-sectional study.

Primary Outcomes

Primary outcomes were measured as at least one grade improvement on disability scale after four weeks or the ability to walk unaided or cured. Thirteen out of 17 studies were assessed for the primary outcome. Most studies reported both treatments to be equally efficacious in improving primary outcomes.

Secondary Outcomes

Secondary outcomes were measured in terms of duration of hospitalization and requirement of MV (ICU stay duration length), the time required to improve by at least one functional grade, and, the ability to regain the capacity for independent locomotion (able to walk unaided or recovered completely). Thirteen out of the 17 studies assessed for secondary outcomes. No significant difference was noted in secondary outcomes among both groups. In children, the secondary outcomes were better in the PE group.

Complications

Both treatment groups were associated with complications. Complications varied from minor problems such as correctable hypotension, hypoalbuminemia, hemolysis, and vagal reactions to major complications like cardiac arrest and pulmonary embolism. Deaths were also reported in a few studies. Adverse effects were more common in the PE group than in the IVIG group.

Other Outcomes (Safety, Cost, and Ease of Use)

Most of the studies reported both treatments to be equally safe and that IVIG was easier to administer. The treatment discontinuity rate was significantly higher among the PE group.

Discussion

The first treatment for GBS was plasma exchange. A randomized experiment published in 1985 (The Guillain-Barré Syndrome Study Group) demonstrated that PE was a highly beneficial treatment [31]. It evolved into the gold standard by which other therapies were evaluated. In 1988, IVIG was first used to treat GBS [32,33]. Subsequent research demonstrated that IVIG was just as effective as PE. Studies showed that steroid therapy was ineffective and played no role in GBS management [34].

This review assessed for efficacies of both the established treatments, i.e., PE and IVIG in terms of primary outcomes, secondary outcomes, safety, complications, and other parameters like cost and ease.

Primary Outcomes

The studies used the Hughes Functional Grading Scale (HFGS) to rate clinical performance as a measure of disability. Scores ranged from 0 to 6, with higher scores indicating a more severe disability as shown in Table 6 [34]. The primary outcomes were defined as being able to walk unassisted or cured, or as having at least one grade improvement on the disability scale after four weeks.

**Table 6:** Hughes Functional Grading Scale (disability scale) HFGS, Hughes Functional Grading Scale

HFGS	
0	Healthy
1	Minor symptoms and signs but fully capable of manual labor
2	Capable of walking ≥10 m without assistance
3	Capable of walking ≥10 m with a walker or support
4	Bedridden or chairbound
5	Requiring assisted ventilation (for some part of the day)
6	Death

Five studies reported that PE is as effective as IVIG in treating GBS, three studies reported that PE was more efficacious than IVIG in treating GBS, and four studies reported IVIG to be more efficacious than PE. Among these studies, a study by Anwer et al. examined treatment efficacy in children and concluded that PE is better than IVIG in improving primary outcomes in children with GBS, and a study by Kuwabara et al. examined treatment efficacies in the IgG anti-GM1-positive subgroup of GBS patients and reported significantly faster clinical recovery after IVIG than PE treatment [24,26]. The findings of our study are consistent with other studies in concluding that IVIG is as efficacious as PE in improving primary outcomes. According to prior research compiled in a Cochrane review, plasma exchange and IVIG are equally effective in improving disability after four weeks [35].

Secondary Outcomes

Thirteen studies assessed the ability to regain independent locomotion/recovery/cure rate: Five studies reported patients with IVIG treatment recovered better and regained independent locomotion earlier than those patients on PE, four studies concluded that both treatments showed no significant difference in recovery or time for regaining independent locomotion, and three studies by Saad et al., answer et al., and El-Bayoumi et al. reported that ability to regain independent locomotion and recovery was better in children with PE treatment [16,24,17]. Similarly, a study by Ye et al. reported that PE therapy can aid with early patient rehabilitation and has a more curative effect by improving symptoms [27].

Ten studies assessed for need and duration of MV: Five studies reported decreased need and duration of MV in patients who received IVIG, two studies reported no significant difference in need and duration of MV, and the studies by Saad et al. and El-Bayoumi et al. reported that duration of MV was significantly less in PE group children, whereas the study by Elahi et al. reported that mean duration of MV stay was significantly higher among children receiving plasma exchange [16,17,28].

Eight studies assessed for length/duration of hospital stay: Five studies reported that the duration of hospital stay was significantly less in the IVIG treatment group, one study by El-Bayoumi et al. reported no significant difference in PICU stay among both treatment groups, and a study by Saad et al. reported that in children duration of hospital stay was less among PE group, whereas a study by Elahi et al. reported that higher length of ICU stay was noted among children receiving plasma exchange [16,17,28].

In our analysis, not much significant difference was noted among the two treatment modalities in improving secondary outcomes, IVIG was found to be slightly superior to PE in reducing the need for MV and duration of hospitalization. However, the secondary outcomes in children were slightly better in the PE group.

Complications

There were no notable adverse effects linked to either of the treatments, according to El Bayoumi et al. [17]. According to van der Merche et al., there were 39 problems in the immune globulin group and 68 in the plasma-exchange group. Patients in the plasma-exchange group experienced multiple complications at a significantly higher rate (16 patients versus five in the immune globulin group; P<0.01) [30]. 20% of the IVIG patients in the trial died, according to Anwer et al. [24]. Two individuals treated with PE had vagal reactions, while patients treated with IVIG did not report any side effects according to Rovasia et al. [22]. According to Ashalatha et al. in the PE group, there were five patients with serious complications: three had deep hypotension that required treatment to be stopped, and two patients passed away - one from a cardiac arrest and the other from acute pulmonary emboli. The plasma exchange group also experienced minor problems, including correctable hypotension in four patients, hypoalbuminemia in two patients, and hemolysis with jaundice in one patient. There were no complications in the IVIG group [20]. Beydoun et al. reported that PE patients experienced increased in-hospital death with a 2.78 odds ratio when compared to IVIG patients [19]. Infections and hypotension occurred at comparable rates in both groups, according to Sudulanguta et al. [23]. In the PSGBS Study Group (1997) trial, after 48 weeks, trial 21 out of 129 IVIG participants and trial 19 out of 114 PE participants were dead or so disabled that they needed assistance to walk (non-significant difference) [25].

Adverse effects and major complications were noted slightly more in the PE group (non-significant); previous research studies have shown that PE is associated with an increased risk of adverse effects and post-treatment worsening symptoms [36].

Other Outcomes (Safety, Cost, and Ease of Use)

Mallick et al. and Saad et al. reported that patients treated with PE had lowered hospitalization costs due to far shorter ICU stays and shorter periods of MV than patients treated with IVIG [18,16]. Beydoun et al. reported that patients who received PE had to be in the hospital for an average of 7.5 days longer and spent $46000 more during their stay [19]. Kishore et al. reported that compared to IVIG, PE management is less expensive. IVIG costs about $2400, while PE costs about $1300 [21]. Sudulanguta et al. reported that across all hospitals in the study, the costs of plasma exchange were substantially lower than those of IVIG [23]. Rodsapert et al. reported that IVIG had a higher unit cost than the other treatment; the difference in unit costs was significant (p-value: <0.05) [15]. PSGBS Study Group (1997) found a statistically significant difference in the number of participants who had their treatment discontinued by at least 25% (18 out of 121 PE and three out of 130 IVIG) [25]. In the Dutch trial, 12 out of 73 participants discontinued one or more PE sessions, but all 74 participants who were assigned to the IVIG treatment completed the courses according to the protocol [30].

The Cost of IVIG treatment was more than PE treatment in our analysis. Similar findings were noted in a previous study in which the direct costs of IVIG therapy were twice as high as PE for GBS patients [37]. It was also observed that IVIG is more user-friendly and has a much lower patient discontinuation rate than PE treatment. The expected extremely significant difference was due to the simplicity of administering IVIG as opposed to PE, which necessitates access to two veins (one of which must tolerate high flow volumes and frequently involves the installation of a central venous line), a PE machine, and specially trained people. IVIG requires access to just one peripheral vein and does not require any specialized tools or personnel with specialized training [35].

The results of our study are consistent with the studies by Zaki et al. and Hughes et al. in concluding that IVIG and PE have similar curative effects [35,38].

Limitations

The review we conducted had several limitations, including the presence of high heterogeneity in the analysis of both primary and secondary outcomes, particularly in relation to duration of hospitalization, ventilation duration, time for regaining independent locomotion, and complications. The studies included in the review had diverse sample sizes. Furthermore, the review incorporated a study involving patients who were administered different IVIG dosages, and the volumes of PE varied among the studies, all contributing to the heterogeneity. We only included data available in English. This may have led to the exclusion of studies relevant to our study.

## Conclusions

IVIG is equally efficacious as PE in improving primary outcomes and secondary outcomes. IVIG showed a slight advantage over PE in reducing the need for MV and hospital stay duration. However, in children, PE had a slight edge over IVIG in improving secondary outcomes. PE was associated with a slightly higher risk of adverse events and post-treatment worsening symptoms compared to IVIG. IVIG is considered more user-friendly with a significantly lower patient discontinuation rate compared to PE. IVIG treatment was found to be significantly more expensive than PE.
